# Comparative Sensitivity Analysis of Muscle Activation Dynamics

**DOI:** 10.1155/2015/585409

**Published:** 2015-08-31

**Authors:** Robert Rockenfeller, Michael Günther, Syn Schmitt, Thomas Götz

**Affiliations:** ^1^Institut für Mathematik, Universität Koblenz, 56070 Koblenz, Germany; ^2^Institut für Sport- und Bewegungswissenschaft, Universität Stuttgart, Allmandring 28, 70569 Stuttgart, Germany; ^3^Institut für Sportwissenschaft, Lehrstuhl für Bewegungswissenschaft, Friedrich-Schiller-Universität, Seidelstraße 20, 07749 Jena, Germany; ^4^Stuttgart Research Centre for Simulation Technology, Pfaffenwaldring 7a, 70569 Stuttgart, Germany

## Abstract

We mathematically compared two models of mammalian striated muscle activation dynamics proposed by Hatze and Zajac. Both models are representative for a broad variety of biomechanical models formulated as ordinary differential equations (ODEs). These models incorporate parameters that directly represent known physiological properties. Other parameters have been introduced to reproduce empirical observations. We used sensitivity analysis to investigate the influence of model parameters on the ODE solutions. In addition, we expanded an existing approach to treating initial conditions as parameters and to calculating second-order sensitivities. Furthermore, we used a global sensitivity analysis approach to include finite ranges of parameter values. Hence, a theoretician striving for model reduction could use the method for identifying particularly low sensitivities to detect superfluous parameters. An experimenter could use it for identifying particularly high sensitivities to improve parameter estimation. Hatze's nonlinear model incorporates some parameters to which activation dynamics is clearly more sensitive than to any parameter in Zajac's linear model. Other than Zajac's model, Hatze's model can, however, reproduce measured shifts in optimal muscle length with varied muscle activity. Accordingly we extracted a specific parameter set for Hatze's model that combines best with a particular muscle force-length relation.

## 1. Introduction

Scientific knowledge is gained by an interplay between quantitative real world measurements of physical, chemical, or biological phenomena and the development of mathematical models for understanding the dynamical processes behind. In general, such phenomena are determined as spatiotemporal patterns of physical measures (state variables). Modelling consists of distinguishing the surrounding world from the system that yields the phenomena and formulating a mathematical description of the system, a model, that can predict values of the state variables. The calculations depend on model parameters and often on giving measured input variables. By changing parameter values and analysing the resulting changes in the values of the state variables, the model may then be used as a predictive tool. This way, the model's validity can be verified. If the mathematical model description is moreover derived from first principles, the model has the potential to explain the phenomena in a causal sense.

Calculating the sensitivities of a model's predicted output, that is, the system's state variables, with respect to model parameters is a means of eliminating redundancy and indeterminacy from models and thus helps to identify valid models. Sensitivity analyses can be helpful both in model-based experimental approaches and in purely theoretical work. A modelling theoretician could be looking for parameters to which all state variables are nonsensitive. Such parameters might be superfluous. An experimenter may inspect the model that represents his working hypothesis and analyse which of the model's state variables are specifically sensitive to a selected parameter. Hence, the experimenter would have to measure exactly this state variable to identify the value of the selected parameter.

In a biomechanical study Scovil and Ronsky [1] applied sensitivity analysis to examine the dynamics of a mechanical multibody system: a runner's skeleton coupled to muscle activation-contraction dynamics. They calculated specific sensitivity coefficients in three slightly different ways. A sensitivity coefficient is the difference quotient calculated from dividing the change in a state variable by the change in a model parameter value, evaluated in a selected system state [2]. The corresponding partial derivative may be simply called “sensitivity.” Therefore, a sensitivity function is the time evolution of a sensitivity [2]. Accordingly, Lehman and Stark [2] had proposed a more general and unified approach than Scovil and Ronsky [1], which allows systematically calculating the sensitivities of any dynamical system described in terms of ordinary differential equations. As an example for sensitivity functions, Lehman and Stark [2] had applied their proposed method to a muscle-driven model of saccadic eye movement. By calculating a percentage change in a state variable value per percentage change in a parameter value, all sensitivities can be made comprehensively comparable, even across models.

A sensitivity as defined so far is of first order. Methodically, we aim at introducing a step beyond, namely, at calculating second order sensitivities. These measures are suited to quantify how much the sensitivity of a state variable with respect to one model parameter depends on changing another parameter. By analysing second order sensitivities, the strength of their interdependent influence on model dynamics can be determined. In addition to this so-called local sensitivity analysis, we will take the whole parameter variability into account by calculating global sensitivities according to Chan et al. [3] and Saltelli and Chan [4]. This approach allows translating the impact of one parameter on a state variable into a parameter's importance, by completely comprising its interdependent influence in combination with all other parameters' sensitivities.

In this study, we will apply the sensitivity analysis to models that predict how the activity of a muscle (its chemical state) changes when the muscle is stimulated by neural signals (electrical excitation). Such models are used for simulations of muscles' contractions coupled to their activation dynamics. Models for coupled muscular dynamics are often part of neuromusculoskeletal models of biological movement systems. In particular, we want to try and rate two specific model variants of activation dynamics formulated by Zajac [5] and by Hatze [6]. As a first result, we present an example of a simplified version of the Zajac [5] model, in which sensitivity functions can in fact be calculated in closed form. Subsequently we calculate the sensitivities numerically with respect to all model parameters in both models, aiming at an increased understanding of the influence of changes in model parameters on the solutions of the underlying ordinary differential equations (ODEs). Additionally, we discuss which of both models may be physiologically more accurate. The arguments come from a mixture of three different aspects: sensitivity analysis, others' experimental findings, and an additional attempt to best fit different combinations of activation dynamics and force-length relations of the contractile element (CE) in a muscle to known data on shifts in optimal CE length with muscle activity [7].

## 2. Two Models for Muscle Activation Dynamics

Macroscopically, a muscle fibre or an assembly thereof, a muscle belly, is often mapped mathematically by a one-dimensional massless thread called “contractile component” or “contractile element” (CE) [8–12]. Its absolute length is *ℓ*_CE_ which may be normalised to the optimal fibre length *ℓ*_CE,opt_ by *ℓ*_CErel_ = *ℓ*_CE_/*ℓ*_CE,opt_. In macroscopic muscle models, the CE muscle force is usually modelled as a function of a force-(CE-)length relation, a force-(CE-)velocity relation, and (CE-)activity *q*. Commonly the muscle activity *q* represents the number of attached cross-bridges within the muscle, normalised to the maximum number available (*q*_0_ ≤ *q* ≤ 1). It can also be considered as the concentration of bound Ca^2+^-ions in the muscle sarcoplasma relative to its physiological maximum. The parameter *q*_0_ represents the minimum activity that is assumed to occur without any stimulation [6].

We analyse two different formulations of muscle activation dynamics, that is, the time (its symbol: *t*) evolution of muscle activity *q*(*t*). One formulation of muscle activation dynamics was suggested by Zajac [5], which we modified slightly to take *q*_0_ into account:

(1) q˙Z=1τ·(1−q0)·σ·1−q0−σ·1−β·qZ−q0      −β·qZ−q0,

with the initial condition *q*_*Z*_(0) = *q*_*Z*,0_. In this context, *σ* is supposed to represent the (electrical) stimulation of the muscle, being a parameter for controlling muscle dynamics. It represents the output of the nervous system's dynamics applied to the muscle which in turn interacts with the skeleton, the body mass distribution, the external environment, and therefore the nervous system in a feedback loop. Electromyographic (EMG) signals can be seen as a compound of such neural stimulations collected in a finite volume (being the input to a number of muscle fibres) over a frequency range and coming from a number of (moto-)neurons. The parameter *τ* denotes the activation time constant, and *β* = *τ*/*τ*_deact_ is the ratio of activation to deactivation time constants (deactivation boost).

An alternative formulation of muscle activation dynamics was introduced by Hatze [6]:

(2) $$\begin{array}{c} \dot{\gamma }=m\cdot \left(\sigma -\gamma \right). \end{array}$$

We divided the original equation from Hatze [6] by the parameter *c* = 1.37 · 10^−4^ mol/L which represents the maximum concentration of free Ca^2+^-ions in the muscle sarcoplasma. Thus, the values of the corresponding normalised concentration are 0 ≤ *γ* ≤ 1. The activity is finally calculated by the function

(3) $$\begin{array}{c} q_{H}\left(\gamma ,l_{\text{CErel}}\right)=\frac{q_{0}+{\left[\rho \left(l_{\text{CErel}}\right)\cdot \gamma \right]}^{\nu }}{1+{\left[\rho \left(l_{\text{CErel}}\right)\cdot \gamma \right]}^{\nu }}, \end{array}$$

and the parameter *c* is shifted to the accordingly renormalised function

(4) $$\begin{array}{c} \rho \left(l_{\text{CErel}}\right)=\rho_{c}\cdot \frac{l_{\rho }-1}{l_{\rho }/l_{\text{CErel}}-1}, \end{array}$$

with *ρ*_*c*_ = *c* · *ρ*_0_ and *ℓ*_*ρ*_ = 2.9. Two cases have been suggested by Hatze [13]: *ρ*_0_ = 6.62 · 10^4^ L/mol (i.e., *ρ*_*c*_ = 9.10) for *ν* = 2 and *ρ*_0_ = 5.27 · 10^4^ L/mol (i.e., *ρ*_*c*_ = 7.24) for *ν* = 3, which have been applied in the literature [7, 8, 14, 15]. By substituting (2) and (3) into ${\dot{q}}_{H}=dq_{H}(\gamma ,\ell_{\text{CErel}})/d\gamma \cdot \dot{\gamma }$ and resubstituting the inverse of (3) afterwards, Hatze's formulation of an activation dynamics can be transformed into a nonlinear differential equation directly in terms of the activity:

(5) q˙H=ν·m1−q0·σ·ρ(lCErel)·1−qH1+1/ν·qH−q01−1/ν    qH−q01−1/ν−(1−qH)·(qH−q0),

with the initial condition *q*_*H*_(0) = *q*_*H*,0_.

The solutions *q*_*Z*_(*t*) and *q*_*H*_(*t*) of both formulations of activation dynamics (1) and (5) can now be directly compared by integrating them with the same initial condition *q*_*Z*,0_ = *q*_*H*,0_ using the same stimulation *σ*.

## 3. Local First and Second Order Sensitivity of ODE Systems regarding Their Parameters

Let *Ω*⊆*ℝ* × *ℝ* ^*M*^ × *ℝ* ^*N*^ and *f* : *Ω* → *ℝ* ^*M*^. We then consider a system of ordinary, first order initial value problems (IVP):

(6) $$\begin{array}{c} \dot{Y}=f\left(t,Y\left(t,\Lambda \right),\Lambda \right),\;\;Y\left(0\right)=Y_{0}, \end{array}$$

where *Y*(*t*) = (*y*_1_(*t*), *y*_2_(*t*),…, *y*_*M*_(*t*)) denotes the vector of state variables, *f* = (*f*_1_, *f*_2_,…, *f*_*M*_) the vector of right hand sides of the ODE, and Λ = {*λ*_1_, *λ*_2_,…, *λ*_*N*_} the set of parameters which the ODE depends on. The vector of initial conditions is abbreviated by

(7) Y0=y10,y20,…,yM0=y1,0,y2,0,…,yM,0=Y0.

The first order sensitivity of the solution *Y*(*t*, Λ) with respect to the parameter set Λ is defined as the matrix

(8) S(t,Λ)=Sikt,Λi=1,…,N,k=1,…,M,with  Sik(t,Λ)=ddλiyk(t,Λ).

Simplifying, we denote *Y* = *Y*(*t*, Λ), *f* = *f*(*t*, *Y*, Λ), and *S*_*ik*_ = *S*_*ik*_(*t*, Λ) but keep the dependencies in mind. Because the solution *Y*(*t*) might only be gained numerically rather than in a closed-form expression, we have to apply the well-known theory of sensitivity analysis as stated in Vukobratovic [16], Dickinson and Gelinas [17], Lehman and Stark [2], and ZivariPiran [18]. Differentiating (8) with respect to *t* and applying the chain rule yield

(9) ddtSik=d2dtdλiyk=d2dλidtyk=ddλifk=ddλiY·∂∂Yfk+∂∂λifk,

with ∂/∂*Y* being the gradient of state variables. Hence we obtain the following ODE for the first order solution sensitivity:

(10) $$\begin{array}{c} {\dot{S}}_{ik}=\overset{M}{\underset{l=1}{\sum }}S_{il}\cdot \frac{\partial }{\partial y_{l}}f_{k}+\frac{\partial }{\partial \lambda_{i}}f_{k},\;\;S_{ik}\left(0\right)=\frac{\partial }{\partial \lambda_{i}}y_{k,0}=0, \end{array}$$

or in short terms

(11) $$\begin{array}{c} \dot{S}=S\cdot J+B,\;\;S\left(0\right)=0_{N\times M}, \end{array}$$

where *S* = *S*(*t*) is the *N* × *M* sensitivity matrix and *J* = *J*(*t*) is the *M* × *M* Jacobian matrix with *J*_*kl*_ = (∂/∂*y*_*l*_)*f*_*k*_; furthermore, *B* = *B*(*t*) denotes the *N* × *M*-matrix containing the partial derivatives *B*_*ik*_ = (∂/∂*λ*_*i*_)*f*_*k*_ and 0_*N*×*M*_ denotes the *N* × *M*-matrix consisting of zeros only.

By analogy, the second order sensitivity of *Y*(*t*) with respect to Λ is defined as the following *N* × *N* × *M*-tensor:

(12) $$\begin{array}{c} R\left(t,\Lambda \right)={\left(R_{ijk}\left(t,\Lambda \right)\right)}_{i,j=1,\ldots ,N,k=1,\ldots ,M}, \end{array}$$

with

(13) $$\begin{array}{c} R_{ijk}\left(t,\Lambda \right)=\frac{d}{d\lambda_{i}}S_{jk}=\frac{d}{d\lambda_{j}}S_{ik}=\frac{d^{2}}{d\lambda_{i}d\lambda_{j}}y_{k}=R_{jik}\left(t,\Lambda \right), \end{array}$$

assuming *R*_*ijk*_ = *R*_*jik*_ for all *k* = 1,…, *M*, therefore assuming that the prerequisites of Schwarz theorem (symmetry of the second derivatives) are fulfilled throughout. Differentiating with respect to *t* and applying the chain rule lead to the ODE

(14) R˙ijk=∑l=1MRijl∂∂ylfk+Sil∂∂λjfk+Sjl∂∂λifk+∑l1=1M∑l2=1MSil1Sjl2∂2∂yl1∂yl2fk+∂2∂λi∂λjfk,

with *R*_*ijk*_(0) = 0. For purposes beyond the aim of this paper, a condensed notation introducing the concept of tensor (or Kronecker) products as in ZivariPiran [18] may be helpful. For a practical implementation in MATLAB see Bader and Kolda [19].

Furthermore, if an initial condition *y*_*k*,0_ (see (7)) is considered as another parameter, we can derive a separate sensitivity differential equation by rewriting (6) in its integral form

(15) $$\begin{array}{c} Y\left(t\right)=Y_{0}+\overset{t}{\underset{0}{\int }}f\left(s,Y\left(s\right)\right)ds. \end{array}$$

Differentiating this equation with respect to *Y*_0_ yields

(16) $$\begin{array}{c} S_{Y_{0}}\left(t\right)=\frac{\partial }{\partial Y_{0}}Y\left(t\right)=1+\overset{t}{\underset{0}{\int }}\frac{\partial }{\partial Y}f\cdot \frac{\partial }{\partial Y_{0}}Y\left(s\right)ds \end{array}$$

and differentiating again with respect to *t* results in a homogeneous ODE for each component *S*_*y*_*k*,0__(*t*); namely,

(17) $$\begin{array}{c} {\dot{S}}_{y_{k,0}}\left(t\right)=\overset{M}{\underset{l=1}{\sum }}\frac{\partial }{\partial y_{l}}f_{k}\cdot S_{y_{l,0}},\;\text{with}\;S_{y_{k,0}}\left(0\right)=\frac{\partial }{\partial y_{k,0}}y_{k,0}=1. \end{array}$$

The parameters of our analysed models are supposed to represent physiological processes and bear physical dimensions therefore. For example, *m* and 1/*τ* are frequencies measured in (Hz), whereas *c* is measured in (mol/L). Accordingly, *S*_*τ*_ = (*d*/*dτ*)*q*_*Z*_ would be measured in (Hz) and *S*_*m*_ in (s) (note that our model only consists of *one* ODE and therefore we do not need a second index). Normalisation provides a comprehensive comparison between all sensitivities, even across models. For any parameter, the value *λ*_*i*_ fixed for a specific simulation is a natural choice. For any state variable, we chose its current value *y*_*k*_(*t*) at each point in time of the corresponding ODE solution. Hence, we normalise each sensitivity *S*_*ik*_ = *dy*_*k*_/*dλ*_*i*_ by multiplying it with the ratio *λ*_*i*_/*y*_*k*_(*t*) to get the relative sensitivity

(18) $$\begin{array}{c} {\tilde{S}}_{ik}=S_{ik}\cdot \frac{\lambda_{i}}{y_{k}}. \end{array}$$

A relative sensitivity ${\tilde{S}}_{ik}$ thus quantifies the percentage change in the *k*th state variable value per percentage change in the *i*th parameter value. This applies accordingly to the second order sensitivity

(19) $$\begin{array}{c} {\tilde{R}}_{ijk}=R_{ijk}\cdot \frac{\lambda_{i}\cdot \lambda_{j}}{y_{k}}. \end{array}$$

It can be shown that this method is valid and mathematically equivalent to another common method in which the whole model is nondimensionalised a priori [20]. A nonnormalised model formulation has the additional advantage of usually allowing a more immediate appreciation of and transparent access for experimenters. In the remainder of this paper, we are always going to present and discuss relative sensitivity values normalised that way.

In our model the specific case *M* = 1 applies, so (10) and (14) simplify to the case *k* = 1 (no summation).

## 4. Variance-Based Global Sensitivity Analysis

The differential sensitivity analysis above is called a local method because it does not take the physiological range of parameter values into account. Additionally factoring in such ranges characterises the so-called global methods. The main idea behind most global methods is to include a statistical component to scan the whole parameter space *𝒞* and combine the percentage change in a state variable value per percentage change in a parameter value with the variability of all of the parameters. The parameter space *𝒞* can be seen as a *N*-dimensional cuboid *𝒞* = [*λ*_1_^−^; *λ*_1_^+^] × ⋯×[*λ*_*N*_^−^; *λ*_*N*_^+^], where *λ*_*i*_^−^ and *λ*_*i*_^+^ are the minimal and maximal parameter values and *N* is the number of parameters. We can now fix a certain point $\hat{\Lambda }=({\hat{\lambda }}_{1},\ldots ,{\hat{\lambda }}_{N})\in 𝒞$ and calculate the local gradient of the solution with respect to $\hat{\Lambda }$. The volume of the star-shaped area, investigated by changing only one parameter at once and lying within a ball around $\hat{\Lambda }$, vanishes in comparison to *𝒞* for an increasing number of parameters [21]. For an overview of the numerous methods like ANOVA, FAST, Regression, or Sobol's Indexing, the reader is referred to Saltelli and Chan [4] and Frey et al. [22].

In this section we want to sketch just the main idea of the variance-based sensitivity analysis approach as presented in Chan et al. [3], which is based on Sobol's Indexing. We chose this method because of its transparency and low computational cost. This method aims at calculating two measurands of sensitivity of a state variable with respect to parameter *λ*_*i*_: the variance-based sensitivity function denoted by VBS_*i*_(*t*) and the total sensitivity index function denoted by TSI_*i*_(*t*). The VBS functions give a normalised first order sensitivity quite similar to $\tilde{S}$ from the previous section but include the parameter range. The TSI functions, however, additionally include higher order sensitivities and give a measurand for interdependencies of parameter influences.

A receipt for calculating VBS and TSI is as follows. First of all, set boundaries for all model parameters, either by model assumptions or by literature reference, thus fixing *𝒞*. Secondly, generate two sets of *n* sample points ${\hat{\Lambda }}_{1,j},{\hat{\Lambda }}_{2,j}\in 𝒞$, *j* = 1,…, *n*, suited to represent the underlying probability distribution of each parameter, in our case the uniform distribution. Thirdly, with *i* indicating a parameter, generate 2*nN* sets of new sample points ${\hat{\Lambda }}_{1,j}^{i},{\hat{\Lambda }}_{1,j}^{\sim i}$, *j* = 1,…, *n*, *i* = 1,…, *N*, where ${\hat{\Lambda }}_{1,j}^{i}$ consists of all sample points in ${\hat{\Lambda }}_{1,j}$ except for its *i*th component (parameter value) replaced by the *i*th component of ${\hat{\Lambda }}_{2,j}$. Consequently, ${\hat{\Lambda }}_{1,j}^{\sim i}$ consists of the *i*th component of ${\hat{\Lambda }}_{1,j}$ and every other component taken from ${\hat{\Lambda }}_{2,j}$. Fourthly, evaluate the model from (6) at all of the 2*n*(*N* + 1) sample points ${\hat{\Lambda }}_{1,j},{\hat{\Lambda }}_{2,j},{\hat{\Lambda }}_{1,j}^{i},{\hat{\Lambda }}_{1,j}^{\sim i}$ resulting in a family of solutions.

For this family perform the following calculations.

(1) Compute the variance of the family of all 2*n*(*N* + 1) solutions as a function of time, namely, *V*(*t*). This variance function indicates the general model output variety throughout the whole parameter range.
(2) Compute the variances *V*_*i*_ of the family of *n*(*N* + 1) solutions resulting from an evaluation of the model at all ${\hat{\Lambda }}_{1,j}$ and ${\hat{\Lambda }}_{1,j}^{i}$, that is, for every *j* and *i*. Each *V*_*i*_(*t*) is a function of time and indicates the model output variety if solely the value *λ*_*i*_ of parameter *i* is changed.
(3) Compute the variances *V*_~*i*_ of the family of *n*(*N* + 1) solutions resulting from an evaluation of the model at all ${\hat{\Lambda }}_{1,j}$ and ${\hat{\Lambda }}_{1,j}^{\sim i}$, that is, for every *j* and *i*. Each *V*_~*i*_(*t*) is a function of time and indicates the model output variety if the value of *λ*_*i*_ is fixed, whereas all other parameter values are changed.

Note that the computations in Chan et al. [3] are done using Monte-Carlo integrals as an approximation. The VBS and TSI can be finally calculated as

(20) $$\begin{array}{c} {\text{VBS}}_{i}\left(t\right)=\frac{V_{i}\left(t\right)}{V\left(t\right)},\;\;{\text{TSI}}_{i}\left(t\right)=1-\frac{V_{\sim i}\left(t\right)}{V\left(t\right)}. \end{array}$$

**Figure 1:**
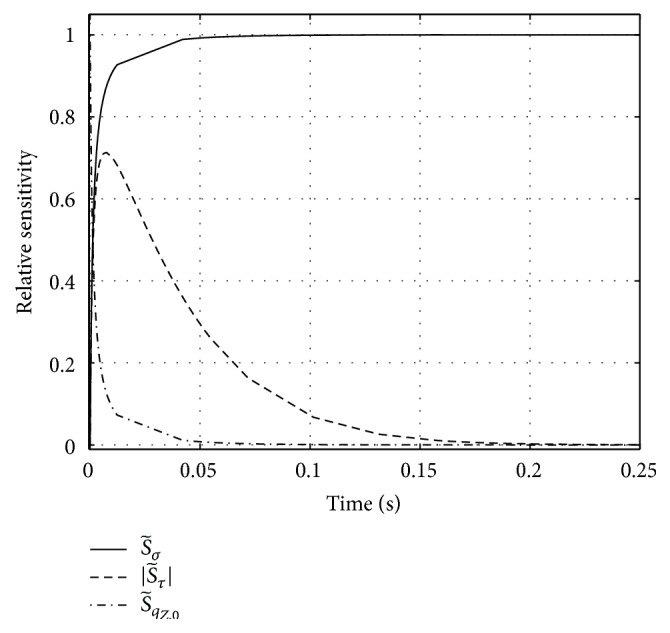
Relative sensitivities ${\tilde{S}}_{i}$ with respect to the three parameters in the simplified formulation (22) of Zajac's activation dynamics (1). Parameters: stimulation *σ* (see (25): solid line), activation time constant *τ* (see (26): dashed line), and initial activation *q*_*Z*,0_ (see (27): dash-dotted line). Note that ${\tilde{S}}_{\tau }$ is negative, but for reasons of comparability we have plotted its absolute value. Parameter values are *σ* = 1, *τ* = 1/40 s = 0.025 s, and *q*_*Z*,0_ = 0.05. Because ODE (22) for *q*_*Z*_^sp^ is equivalent to Hatze's ODE (2) for the free Ca^2+^-ion concentration, *γ*, we can identify the sensitivity of 1/*τ* with that of *m*.

The normalisation entails additional properties of VBS and TSI (see [3, Figure 1]):

(21) $$\begin{array}{c} \overset{N}{\underset{i=1}{\sum }}{\text{VBS}}_{i}\left(t\right)\leq 1,\;\;\overset{N}{\underset{i=1}{\sum }}{\text{TSI}}_{i}\left(t\right)\geq 1. \end{array}$$

In other words, VBS_*i*_(*t*) gives the normalised global first order sensitivity function of the solution with respect to *λ*_*i*_ in relation to the model output range. Accordingly, TSI_*i*_(*t*) quantifies a relative impact of the variability in parameter *λ*_*i*_ on the model output, factoring in the interdependent influence in combination with all other parameters' sensitivities. Chan et al. [3] suggested to denote the TSI_*i*_(*t*) value as the “importance” of *λ*_*i*_.

## 5. An Analytical Example for Local Sensitivity Analysis including a Link between Zajac's and Hatze's Formulations

By further simplifying Zajac's formulation of an activation dynamics (1) through assuming a deactivation boost *β* = 1 (activation and deactivation time constants are equal) and a basic activity *q*_0_ = 0, we obtain a linear ODE for this specific case *q*_*Z*_^sp^, which is equivalent to Hatze's equation (2) modelling the time evolution of the free Ca^2+^-ion concentration:

(22) $$\begin{array}{c} {\dot{q}}_{Z}^{\text{sp}}=\frac{1}{\tau }\left(\sigma -q_{Z}^{\text{sp}}\right),\;\;q_{Z}^{\text{sp}}\left(0\right)=q_{Z,0}. \end{array}$$

By analysing this specific case, we aim at making the above described sensitivity analysis method more transparent for the reader. Solving (22) yields

(23) $$\begin{array}{c} q_{Z}^{\text{sp}}\left(t\right)=\sigma \cdot \left(1-e^{-t/\tau }\right)+q_{Z,0}\cdot e^{-t/\tau } \end{array}$$

depending on just two parameters *σ* (stimulation: control parameter) and *τ* (time constant of activation: internal parameter) in addition to the initial value *y*_0_ = *q*_*Z*,0_. The solution *q*_*Z*_(*t*) equals the *σ* value after about *τ*.

We apply the more generally applicable, implicit methods (10) and (17) to determine the derivatives of the solution with respect to the parameters (the sensitivities), although we already know solution (23) in a closed form. Hence, for the transparency of our method, we calculate the gradient of the right hand side *f*(*q*_*Z*_^sp^, *σ*, *τ*) of ODE (22)

(24) $$\begin{array}{c} \frac{\partial }{\partial q_{Z}^{\text{sp}}}f=-\frac{1}{\tau },\;\;\frac{\partial }{\partial \sigma }f=\frac{1}{\tau },\\ \frac{\partial }{\partial \tau }f=-\frac{\sigma -q_{Z}^{\text{sp}}}{\tau^{2}}=\frac{q_{Z,0}-\sigma }{\tau^{2}}e^{-t/\tau } \end{array}$$

and insert these partial derivatives into (10) and (17). Solving the respective three ODEs for the three parameters (*σ*, *τ*, and *q*_*Z*,0_) and normalising them according to (18) give the relative sensitivities of *q*_*Z*_^sp^ with respect to *σ*, *τ*, and *q*_*Z*,0_ as functions of time (see Figure 1):

(25) $$\begin{array}{c} {\tilde{S}}_{\sigma }\left(t\right)=\left(1-e^{-t/\tau }\right)\cdot \frac{\sigma }{q_{Z}^{\text{sp}}\left(t\right)}=\frac{\sigma \cdot \left(e^{t/\tau }-1\right)}{\sigma \cdot \left(e^{t/\tau }-1\right)+q_{Z,0}}, \end{array}$$

(26) S~τt=(qZ,0−σ)·tτ2e−t/τ·τqZsp(t)=t·(qZ,0−σ)τ·[σ·(et/τ−1)+qZ,0],

(27) $$\begin{array}{c} {\tilde{S}}_{q_{Z,0}}\left(t\right)=e^{-t/\tau }\cdot \frac{q_{Z,0}}{q_{Z}^{\text{sp}}\left(t\right)}=\frac{q_{Z,0}}{\sigma \cdot \left(e^{t/\tau }-1\right)+q_{Z,0}}. \end{array}$$

A straightforward result is that the time constant *τ* has its maximum effect on the solution (Figure 1, see ${\tilde{S}}_{\tau }(t)$) at time *t* = *τ*. In case of a step in stimulation, the sensitivity ${\tilde{S}}_{\tau }(t)$ vanishes in the initial situation and exponentially approaches zero again after a few further multiples of the typical period *τ*. Note that ${\tilde{S}}_{\tau }(t)$ is negative, which means that an increase in *τ* decelerates activation. Thus, for a fixed initial value *q*_*Z*,0_, the solution value *q*_*Z*_(*t*) decreases at a given point in time if *τ* is increased. After a step in stimulation *σ*, the time in which the solution *q*_*Z*_(*t*) bears some memory of its initial value *q*_*Z*,0_ is equal to the period of being nonsensitive to any further step in *σ* (compare ${\tilde{S}}_{q_{Z,0}}(t)$ to ${\tilde{S}}_{\sigma }(t)$ and (25) to (27)). After about *τ*/2, the sensitivity ${\tilde{S}}_{q_{Z,0}}(t)$ has already fallen to about 0.1 and ${\tilde{S}}_{\sigma }(t)$ to about 0.9 accordingly.

## 6. The Numerical Approach and Results

Typically, biological dynamics are represented by nonlinear ODEs. Therefore, the linear ODE used for describing activation dynamics in the Zajac [5] case (1) is more of an exception. For example, a closed-form solution can be given. Equation (23) is an example as shown in the previous section for the reduced case of nonboosted deactivation (22).

In general, however, nonlinear ODEs used in biomechanical modelling, as the Hatze [6] case (5) for describing activation dynamics, can only be solved numerically. It is understood that any explicit formulation of a model in terms of ODEs allows providing the partial derivatives of their right hand sides *f* with respect to the model parameters in a closed form. Fortunately, this is exactly what is required as part of the sensitivity analysis approach presented in Section 3, in particular in (10).

As an application for applying this approach, we will now present a comparison of both formulations of activation dynamics. The example indicates that the approach may be of general value because it is common practice in biomechanical modelling to (i) formulate the ODEs in closed form and (ii) integrate the ODEs numerically. Adding further sensitivity ODEs for model parameters then becomes an inexpensive enhancement of the procedure used to solve the problem anyway.

For the two different activation dynamics [5, 6], the parameter sets Λ_*Z*_ and Λ_*H*_, respectively, consist of

(28) $$\begin{array}{c} \Lambda_{Z}=\left\{q_{Z,0},\sigma ,q_{0},\tau ,\beta \right\}, \end{array}$$

(29) $$\begin{array}{c} \Lambda_{H}=\left\{q_{H,0},\sigma ,q_{0},m,\rho_{c},\nu ,l_{\rho },l_{\text{CErel}}\right\}, \end{array}$$

including the initial conditions. The numerical solutions for these ODEs were computed within the MATLAB environment (The MathWorks, Natick, USA; version R2013b), using the preimplemented numerical solver *ode*45 which is a Runge-Kutta algorithm of order 5 (for details see [23]).

### 6.1. Results for Zajac's Activation Dynamics: Sensitivity Functions

**Figure 2:**
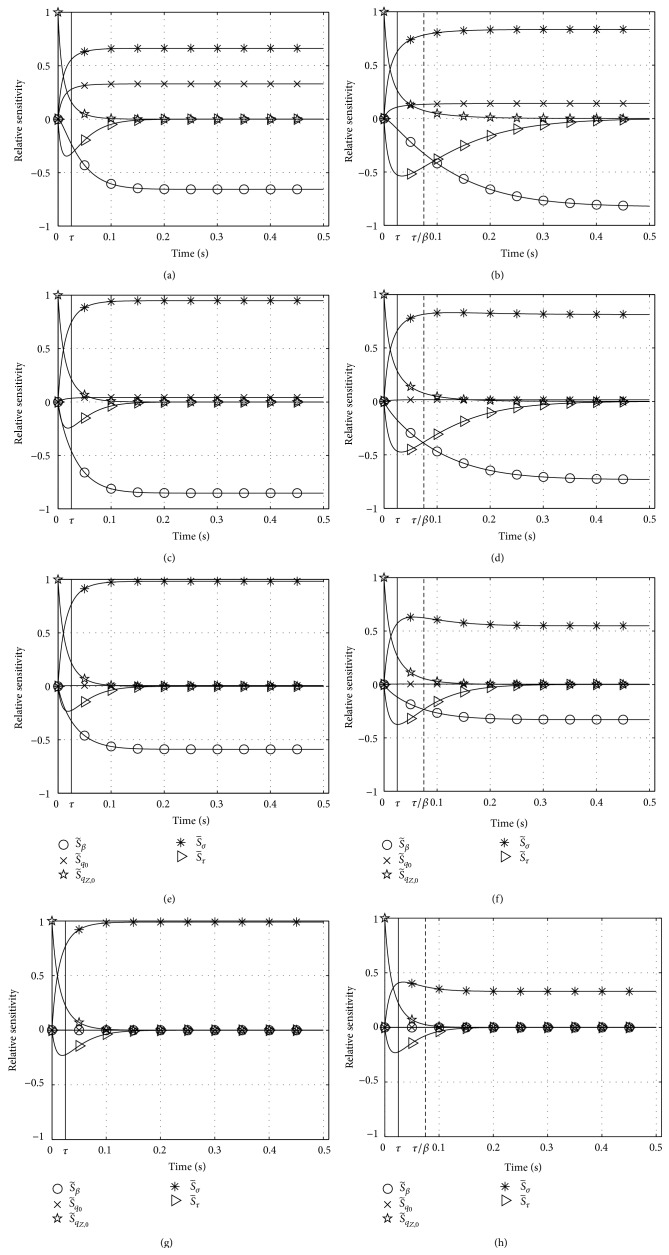
Relative sensitivities ${\tilde{S}}_{i}$ with respect to all parameters *λ*_*i*_ (set Λ_*Z*_ (28)) in Zajac's activation dynamics (1). Parameter values varied from top (i) to bottom (iv) row: (i) *q*_*Z*,0_ = *q*_0_ = 0.005, *σ* = 0.01, (ii) *q*_*Z*,0_ = 0.05, *σ* = 0.1, (iii) *q*_*Z*,0_ = 0.2, *σ* = 0.4, and (iv) *q*_*Z*,0_ = 0.5, *σ* = 1; left column: *β* = 1, right column: *β* = 1/3.

We simulated activation dynamics for the parameter set Λ_*Z*_ (28) leaving two of the values constant (*q*_0_ = 0.005, *τ* = 1/40 s) and varying the other three (initial condition *q*_*Z*,0_, stimulation *σ*, and deactivation boost *β*). The time courses of the relative sensitivities ${\tilde{S}}_{i}(t)$ with respect to all parameters *λ*_*i*_ ∈ Λ_*Z*_ are plotted in Figure 2. In the left column of Figure 2 we used *β* = 1, in the right column *β* = 1/3. Pairs of the parameter values *q*_0_ = 0.005 ≤ *q*_*Z*,0_ ≤ 0.5 and 0.01 ≤ *σ* ≤ 1 are specified in the legend of Figure 2, with increasing values of both parameters from top to bottom.

#### 6.1.1. Relative Sensitivity ${\tilde{S}}_{q_{0}}$

Solutions are nonsensitive to the *q*_0_ choice except if both initial activity and stimulation (also approximating the final activity if *β* = 1 and *σ* ≫ *q*_0_) are very low near *q*_0_ itself.

#### 6.1.2. Relative Sensitivity ${\tilde{S}}_{q_{Z,0}}$

The memory (influence on solution) of the initial value is lost after about 2*τ*, almost independently of all other parameters. This loss in memory is obviously slower than in that case *q*_*Z*,0_ = 0 (initial value) and *σ* = 1 (for *β* = 1 and *q*_0_ = 0 exactly the final value; see Section 5 and Figure 1). In that extreme case, the influence (relative sensitivity) of the lowest possible initial value (*q*_*Z*,0_ = 0) on the most rapidly increasing solution (maximum possible final value: *σ* = 1) is lost earlier.

#### 6.1.3. Relative Sensitivity ${\tilde{S}}_{\tau }$

The influence of the time constant *τ* on the solution is reduced with decreasing difference between initial and final activity values (compare maximum ${\tilde{S}}_{\tau }$ values in Figures 1 and 2) and, no matter the *β* value, with jointly raised levels of initial activity *q*_*Z*,0_ and *σ*, the latter determining the final activity value if *β* = 1. When deactivation is slower than activation (*β* < 1: right column in Figure 2), ${\tilde{S}}_{\tau }$ is higher than in the case *β* = 1, both in its maximum amplitude and for longer times after the step in stimulation, especially at low activity levels (upper rows in Figure 2).

#### 6.1.4. Relative Sensitivity ${\tilde{S}}_{\sigma }$

Across all parameters, the solution in general is most sensitive to *σ*. However, the influence of the deactivation boost parameter *β* is usually comparable. In some situations, this also applies to the activation time constant *τ* (see below). For *β* = 1 (Figure 2, left), the solution becomes a little less sensitive to *σ* with decreasing activity level (${\tilde{S}}_{\sigma }<1$), which reflects that the final solution value is not determined by *σ* alone but by *q*_0_ > 0 and *β* ≠ 1 as much. If deactivation is much slower than activation (*β* = 1/3 < 1: Figure 2, right), we find the opposite to the *β* = 1 case: the more the activity level rises, the lesser *σ* determines the solution. Additionally, stimulation *σ* somehow competes with both deactivation boost *β* and time constant *τ* (see further below). Using the term “compete” illustrates the idea that any single parameter should have an individual interest in influencing the dynamics as much as possible in order not to be considered superfluous.

#### 6.1.5. Relative Sensitivity ${\tilde{S}}_{\beta }$

Sensitivity with respect to *β* generally decreases with increasing activity *q*_*Z*,0_ and stimulation *σ* levels. It vanishes at maximum stimulation *σ* = 1.

#### 6.1.6. Relative Sensitivities ${\tilde{S}}_{\sigma }$, ${\tilde{S}}_{\beta }$, ${\tilde{S}}_{\tau }$

At submaximal stimulation levels *σ* < 1, the final solution value is determined to almost the same degree by stimulation *σ* and deactivation boost *β*, yet with opposite tendencies (${\tilde{S}}_{\sigma }>0$, ${\tilde{S}}_{\beta }<0$). As explained, both parameters compete for their impact on the final solution value. Only at maximum stimulation *σ* = 1 (lowest row in Figure 2), this parameter competition is resolved in favour of *σ*. In this specific case, *β* does not influence the solution at all. For *β* = 1 the competition about influencing the solution is intermittently but only slightly biased by *τ*: sensitivity ${\tilde{S}}_{\tau }$ peaks at comparably low magnitude around *t* = *τ*. This *τ* influence comes likewise intermittently at the cost of *β* influence: the absolute value of ${\tilde{S}}_{\beta }$ rises a little slower than ${\tilde{S}}_{\sigma }$. In the case *β* < 1, this competition becomes more differentiated and spread out in time. Again at submaximal stimulation and activity levels, the absolute value of ${\tilde{S}}_{\tau }$ is lower than that of ${\tilde{S}}_{\sigma }$ but higher than that of ${\tilde{S}}_{\beta }$, making all three parameters *σ*, *β*, and *τ* compete to comparable degrees for an impact on the solution until about *t* = 4*τ*. Also, ${\tilde{S}}_{\tau }$ does not vanish before about *t* = 10*τ*.

### 6.2. Results for Hatze's Activation Dynamics: Sensitivity Functions

**Figure 3:**
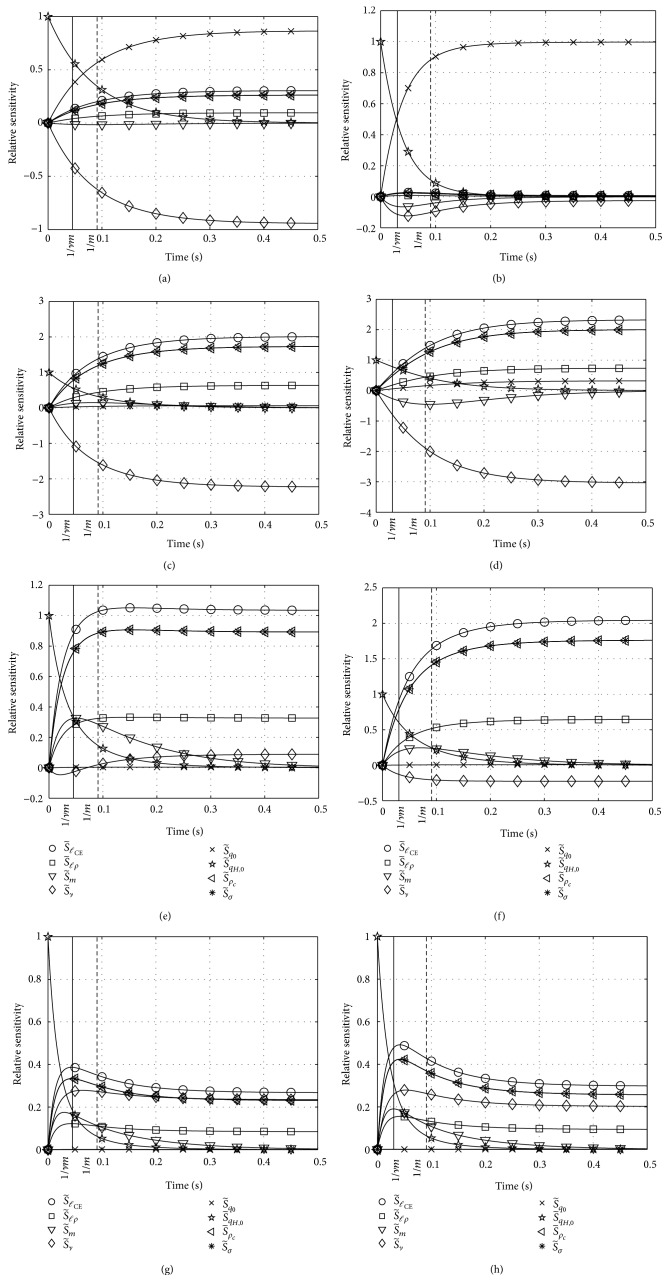
Relative sensitivities ${\tilde{S}}_{i}$ with respect to all parameters *λ*_*i*_ (set Λ_*H*_ (29)) in Hatze's activation dynamics (5). Parameter values varied from top (i) to bottom (iv) row: (i) *q*_*H*,0_ = *q*_0_ = 0.005, *σ* = 0.01, (ii) *q*_*H*,0_ = 0.05, *σ* = 0.1, (iii) *q*_*H*,0_ = 0.2, *σ* = 0.4, and (iv) *q*_*H*,0_ = 0.5, *σ* = 1; left column: *ν* = 2, *ρ*_*c*_ = 9.10, right column: *ν* = 3, *ρ*_*c*_ = 7.24.

We also simulated activation dynamics for the parameter set Λ_*H*_ (29), leaving now four of the values constant (*q*_0_ = 0.005, *m* = 10 1/s, *ℓ*_*ρ*_ = 2.9, *ℓ*_CErel_ = 1) and again varying three others (initial condition *q*_*Z*,0_, stimulation *σ*, and nonlinearity *ν*), keeping in mind that the eighth parameter (*ρ*_*c*_) is assumed to depend on *ν*. Time courses of the relative sensitivities ${\tilde{S}}_{i}(t)$ with respect to all parameters *λ*_*i*_ ∈ Λ_*H*_ are plotted (see Figure 3). In the left column of Figure 3, *ν* = 2, *ρ*_*c*_ = 9.10 is used, in the right column *ν* = 3, *ρ*_*c*_ = 7.24. Here, the same pairs of the parameter values (*q*_0_ = 0.005 ≤ *q*_*Z*,0_ ≤ 0.5 and 0.01 ≤ *σ* ≤ 1, increasing from top to bottom; see legend of Figure 3) are used as in Section 6.1 (Figure 2).

Hatze's activation dynamics (5) are nonlinear unlike Zajac's activation dynamics (1). This nonlinearity manifests particularly in a changeful influence of the parameter *ν*. Additionally, the parameter *m* is just roughly comparable to the inverse of the exponential time constant *τ* in Zajac's linear activation dynamics.

#### 6.2.1. Relative Sensitivity ${\tilde{S}}_{m}$

In Zajac's linear differential equation (1), *τ* establishes a distinct time scale independent of all other parameters. The parameter *m* in Hatze's activation dynamics (5) is just formally equivalent to the reciprocal of *τ*: the sensitivity ${\tilde{S}}_{m}$ does not peak stringently at *t* = 1/*m* = 0.1 s but rather diffusely between about 0.05 s and 0.1 s in both of the cases *ν* = 2 and *ν* = 3. At first this is not surprising because the scaling factor in Hatze's dynamics is *ν* · *m* rather than just *m*. However, *ν* · *m* does not fix an invariant time scale for Hatze's nonlinear differential equation. This fact becomes particularly prominent at extremely low activity levels for *ν* = 2 (Figure 3, left, top row) and up to moderately submaximal activity levels for *ν* = 3 (Figure 3, right, top two rows). Here, ${\tilde{S}}_{m}$ is negative, which means that increasing the parameter *m* results in less steeply increasing activity. This observation is counterintuitive to identifying *m* with a reciprocal of a time constant like *τ*. Rather than being expected from the product *ν* · *m*, the exponent *ν* does not linearly scale the time behaviour because ${\tilde{S}}_{m}$ peaks do not occur systematically earlier in the *ν* = 3 case as compared to *ν* = 2.

#### 6.2.2. Relative Sensitivity ${\tilde{S}}_{q_{H,0}}$

Losing the memory of the initial condition confirms the analysis of time behaviour based on ${\tilde{S}}_{m}$. At high activity levels (Figure 3, bottom row), Hatze's activation dynamics loses memory at identical time horizons (no matter the *ν* value) seemingly slower for higher *ν* at intermediate levels (Figure 3, two middle rows) and clearly faster at very low levels (Figure 3, top row). The parameter *m* still does roughly determine the time horizon in which the memory of the initial condition *q*_*H*,0_ is lost and the influence of all other parameters is continuously switched on from zero influence at *t* = 0.

#### 6.2.3. Relative Sensitivity ${\tilde{S}}_{q_{0}}$

As in Zajac's dynamics the solution is generally only sensitive to *q*_0_ at very low stimulation levels *σ* ≈ *q*_0_ (Figure 3, top row). At such levels, the *ν* = 3 case shows the peculiarity that the solution becomes strikingly insensitive to any other parameter than *q*_0_ itself (and *q*_*H*,0_). The time evolution of the solution is more or less determined by just this minimum (*q*_0_) and initial (*q*_*H*,0_) activities, and *m* determining the approximate switching time horizon between both. The *ℓ*_CE_ dependency, constituting a crucial property of Hatze's activation dynamics, is practically suppressed for *ν* = 3 at very low activities and stimulations. In contrast, ${\tilde{S}}_{\ell_{\text{CErel}}}$ remains for *ν* = 2 on a low but still significant level of about a fourth of the three dominating quantities ${\tilde{S}}_{q_{0}}$, ${\tilde{S}}_{q_{H,0}}$, and ${\tilde{S}}_{\nu }$.

#### 6.2.4. Relative Sensitivity ${\tilde{S}}_{\nu }$

The sensitivity with respect to *ν* is extraordinarily high at low activities and stimulations around 0.1, both for *ν* = 2 and for *ν* = 3 (Figure 3, second row from top), additionally at extremely low levels for *ν* = 2 (Figure 3, left, top row). At moderately submaximal levels (Figure 3, third row from top), the solution is influenced with an already inverted tendency (${\tilde{S}}_{\nu }$ changes sign to positive) after around a 1/*m* time horizon for *ν* = 2. However, at these levels the solution is practically insensitive to *ν* for any *ν*. At high levels (Figure 3, bottom row) we find that there is no change in the character of time evolution of the solution, despite the specific value of *ν*. The degree of nonlinearity *ν* is unimportant because the time evolution and the ranking of all other sensitivities are hardly influenced by *ν*. In both cases, the rise in activity is quickened by increasing *ν* (${\tilde{S}}_{\nu }>0$), as opposed to low activity and stimulation levels where rises in activity are slowed down (${\tilde{S}}_{\nu }<0$; see also above).

#### 6.2.5. Relative Sensitivities ${\tilde{S}}_{\sigma }$, ${\tilde{S}}_{\rho_{c}}$, ${\tilde{S}}_{\ell_{\text{CErel}}}$, and ${\tilde{S}}_{\ell_{\rho }}$

Of all the remaining parameters, stimulation *σ*, scaled maximum free Ca^2+^-ion concentration *ρ*_*c*_, relative CE length *ℓ*_CErel_, and the pole *ℓ*_*ρ*_ of the length dependency in Hatze's activation dynamics, the latter has the lowest influence on the solution. The influence characters of all four parameters are yet completely identical. Their sensitivities are always positive and coupled by fixed scaling ratios due to all of them occurring within just one product on the right side of (5). ${\tilde{S}}_{\sigma }$ and ${\tilde{S}}_{\rho_{c}}$ are identical, while the sensitivity with respect to *ℓ*_CErel_ is the highest, with ratios ${\tilde{S}}_{\ell_{\text{CErel}}}/{\tilde{S}}_{\ell_{\rho }}\approx 3$ and ${\tilde{S}}_{\ell_{\text{CErel}}}/{\tilde{S}}_{\sigma }\approx 1.2$. Except at very low activity (where *q*_0_ plays a dominating role) and except for the generally changeful *ν* influence, these are the four parameters that dominate the solution after an initial phase in which the initial activity *q*_*H*,0_ determines its evolution. The parameter *m* does not have a strong direct influence on the solution. As stated above, it defines the approximate time horizon in which the *q*_*H*,0_ influence gets lost and all other parameters' influence is switched on from zero at *t* = 0.

### 6.3. Variance-Based Sensitivity (VBS) and Total Sensitivity Indices (TSI) for Zajac's and Hatze's Activation Dynamics

**Figure 4:**
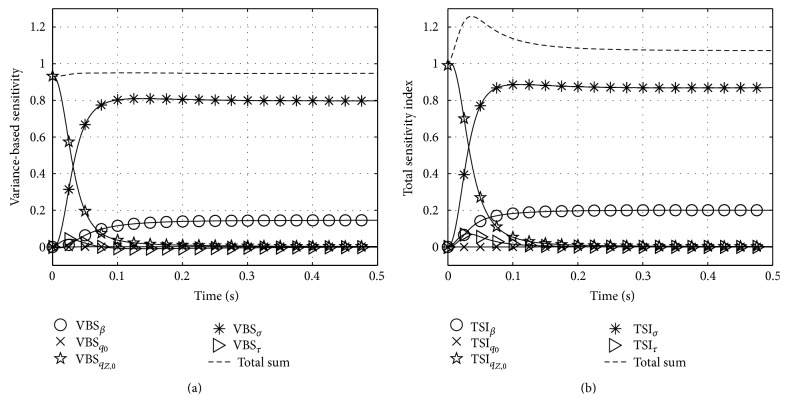
Variance-based sensitivity (a) and total sensitivity index (b) of every parameter of Zajac's activation dynamics equation.

Table 1 pools the lower and upper boundaries for every parameter in Λ_*Z*_ and Λ_*H*_ used in our calculations. We refer to Hatze [24], Zajac [5], or Günther et al. [11] for traceability of our choices. The left hand side of Figure 4 shows the VBS functions of every parameter in Λ_*Z*_ of Zajac's model. The plotted functions can be compared to our previously computed relative first order sensitivity functions from Figure 2: at first sight, ${\tilde{S}}_{q_{Z,0}}$ and VBS_*q*_*Z*,0__ look equal, but the VBS function indicates a slightly increased duration of influence of *q*_*Z*,0_. Regarding *τ*, the VBS function peaks at the same time as ${\tilde{S}}_{\tau }$, but with a smaller amplitude. Likewise, the courses of VBS_*σ*_ and VBS_*β*_ are comparable to ${\tilde{S}}_{\sigma }$ and ${\tilde{S}}_{\beta }$ from the second and third row of Figure 2. The calculated VBS functions in the Zajac case show what would be expected intuitively: a VBS represents a parameter's mean influence averaged over its range of values. Additionally, we plotted the sum of all first order sensitivities. This sum indicates which amount of the total variance is covered by first order sensitivities. The closer the sum to 1 the smaller the impact of the second and higher order sensitivities.

The right hand side of Figure 4 shows the TSI functions of every parameter in Λ_*Z*_ of Zajac's model. Generally, there are only minor deviations of the TSI_*i*_ functions from their counterparts VBS_*i*_. That is, the influence of none of the parameters is significantly enhanced by an interdependent effect in combination with other parameters. According to both analyses, there are just four globally important parameters that govern the system's state throughout the whole examined solution space: the initial condition *q*_*Z*,0_ within a typical time horizon *τ* after a step in *σ*, the new stimulation level *σ* determining activity after about *τ*, the deactivation boost *β* with smaller impact than *σ*, and *τ* determining the time horizon itself.

**Figure 5:**
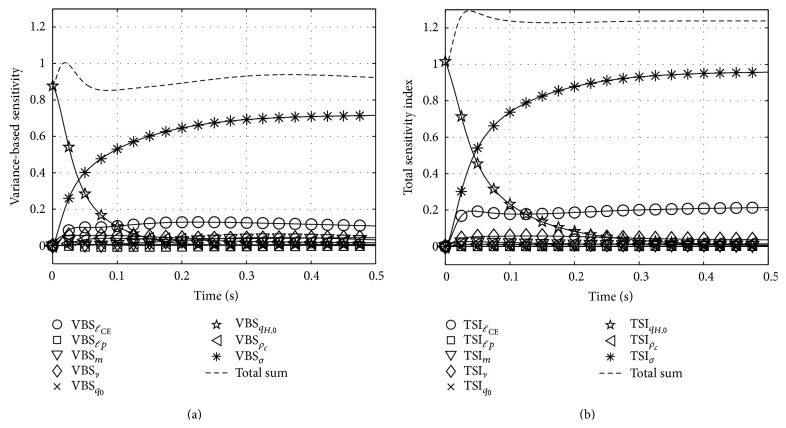
Variance-based sensitivity (a) and total sensitivity index (b) of every parameter of Hatze's activation dynamics equation.

The left hand side of Figure 5 shows the VBS functions of every parameter in Λ_*H*_ of Hatze's model. Very similar to the Zajac case, the calculated VBS seemingly represent to a high degree a parameter's mean influence averaged over its range of values (compare Figure 3). As in the Zajac case, there are four globally important parameters, according to both VBS and TSI analyses. Compared to Zajac's model, the interdependent effect in combination with other parameters (TSI: right hand side of Figure 5) is more pronounced for two parameters: both the stimulation *σ* and the CE length *ℓ*_CE_ importance are distinctly higher than their first order effects as expressed by VBS functions. Furthermore, the time horizon within the initial condition *q*_*H*,0_ has an aftereffect in response to a step in *σ* globally a little higher in VBS as compared to local sensitivity analysis (Figure 3). In addition, the time horizon of *q*_*H*,0_ is clearly enhanced by interdependencies with other parameters (TSI: right hand side of Figure 5).

Altogether, VBS versus TSI analysis substantiate local first and second order sensitivity analyses: for one thing, Hatze's model is more inert against steps in stimulation than Zajac's model. For another thing, the dynamics described by Hatze's model incorporates stronger nonlinear coupling effects from combinations of parameters than Zajac's model. These latter effects are better seen in detail when looking at local sensitivities, that is, analysing just small and selected volumes of the parameter space *𝒞*. In turn, VBS and TSI provide a broad but coarse overview about first and higher order sensitivities of all parameters.

## 7. Consequences, Discussion, and Conclusions

### 7.1. A Bottom Line for Comparing Zajac's and Hatze's Activation Dynamics: Second Order Sensitivities

At first sight, Zajac's activation dynamics [5] is more transparent because it is descriptive in a sense that it captures the physiological behaviour of activity rise and fall in an apparently simple way. It thereto utilises a linear differential equation with well-known properties, allowing for a closed-form solution. It needs only four parameters to describe the Ca^2+^-ion influx to the muscle as a response to electrical stimulation: the stimulation *σ* itself as a control parameter, the time constant *τ* for an exponential response to a step increase in stimulation, a third parameter *β* (deactivation boost) biasing both the rise and fall times, and the saturation value *q*_*Z*_|^*∞*^ of activity which in turn depends on *σ* and the basic activity *q*_0_ being the fourth parameter. The smaller the *β* < 1 is (deactivation slower than activation), the faster the very activity level *q*_*Z*_|_*β*=1_^*∞*^ = *q*_0_ + *σ* · (1 − *q*_0_) is reached, at which saturation would occur for *β* = 1. Saturation for *β* < 1 occurs at a level *q*_*Z*_|_*β*_^*∞*^ = *q*_0_ + (1 − *q*_0_)/(1 − *β* + *β*/*σ*) that is higher than *q*_*Z*_|_*β*=1_^*∞*^. Altogether, in Zajac's as compared to Hatze's activation dynamics, the outcome of setting a control parameter value *σ*, with regard to how fast and at which level the activity saturates, seems easier to be handled by a controller.

A worse controllability of Hatze's activation dynamics [6] may be expected from its nonlinearity, a higher number of parameters, and their interdependent influence on model dynamics. Additionally, Hatze's formulation depends on the CE length *ℓ*_CE_, which makes the mutual coupling of activation with contraction dynamics more interwoven. So, at first sight, Hatze's dynamics seems a less manageable construct for a controller to deal with a muscle as the biological actuator. Regarding the nonlinearity exponent *ν*, solution sensitivity further depends nonmonotonously on activity level, partly even with the strongest influence, partly without any influence. We also found that the solution is more sensitive to its parameters *σ*, *ℓ*_CErel_, and *ℓ*_*ρ*_ than is Zajac's activation dynamics to any of its parameters.

This higher complexity of Hatze's dynamics becomes even more evident by analysing the second order sensitivities (see (13) or (19) for their relative values). They express how a first order sensitivity changes upon variation of any other model parameter. In other words, they are a measure of model entanglement and complexity. Here, we found that the highest values amongst all relative second order sensitivities in Zajac's activation dynamics are about −0.8 (${\tilde{R}}_{\beta \sigma }$) and 1.6 (${\tilde{R}}_{\beta \beta }$). In Hatze's activation dynamics, the highest relative second order sensitivities are those with respect to *ν* or *ℓ*_CErel_ (in particular for *σ*, *ρ*_*c*_, and *ν*, *ℓ*_CErel_ themselves) with maximum values between about −8.0 (${\tilde{R}}_{\ell_{\text{CErel}}\nu }$, ${\tilde{R}}_{\nu \rho_{c}}$) and 13.4 (${\tilde{R}}_{\ell_{\text{CErel}}\ell_{\text{CErel}}}$, ${\tilde{R}}_{\ell_{\text{CErel}}\rho_{c}}$, ${\tilde{R}}_{\ell_{\text{CErel}}\sigma }$, ${\tilde{R}}_{\nu \nu }$ at submaximal activity). That is, they are an order of magnitude higher than in Zajac's activation dynamics.

Yet, we have to acknowledge that Hatze's activation dynamics contains crucial physiological features that go beyond Zajac's description.

### 7.2. A Plus for Hatze's Approach: Length Dependency

It has been established that the length dependency of activation dynamics is both physiological [7] and functionally vital [15] because it largely contributes to low-frequency muscle stiffness. It has also been verified that Hatze's model approach provides a good approximation for experimental data [7]. In that study, *ν* = 3 was used without comparing to the *ν* = 2 case. There seem to be arguments in favour of *ν* = 2 from a mathematical point of view. In particular, the less changeful scaling of the activation dynamics' characteristics down to very low activity and stimulation levels, at which some CE length sensitivity remains, seem to be an advantage when compared to the *ν* = 3 case. Up to this point, we have argued solely mathematically. It is, however, physiological reality that is eventually aimed at. We therefore repeated the model fit done by Kistemaker et al. [7] while now allowing a variation in *ν* and in force-length relations.

### 7.3. An Optimal Parameter Set for Hatze's Activation Dynamics Plus CE Force-Length Relation

Sensitivity analysis allows rating Hatze's approach as an entangled construct. Additionally, Kistemaker et al. [7] decided to choose *ν* = 3 without giving a reason for discarding *ν* = 2. It further seemed that they did not perform an algorithmic optimisation across various submaximal stimulation levels to find a muscle parameter set, which best fits known shifts Δ*ℓ*_CE,opt,submax_ = *ℓ*_CE,opt_ − *ℓ*_CE,opt,submax_ in optimal, submaximal CE length *ℓ*_CE,opt,submax_ at which isometric force *F*_isom_ = *F*_isom_(*q*, *ℓ*_CE_) peaks. Accordingly, it seemed worth performing such an optimisation because *F*_isom_ generally depends on length *ℓ*_CE_ and activity *q*, and the latter may be additionally biased by an *ℓ*_CE_-dependent capability for building up cross-bridges at a given level *γ* of free Ca^2+^-ions in the sarcoplasma, as formulated in Hatze's approach: *F*_isom_(*q*, *ℓ*_CE_) = *F*_max⁡_ · *q*(*γ*, *ℓ*_CE_) · *F*_*ℓ*_(*ℓ*_CE_). Thus, a shift in optimal CE length Δ*ℓ*_CE,opt,submax_ with changing *γ* can occur depending on the specific choices of both the length dependency of activation *q*(*γ*, *ℓ*_CE_) (see (3) and (4)) and the CE's force-length relation *F*_*ℓ*_(*ℓ*_CE_).

Consequently, we searched for optimal parameter sets of Hatze's activation dynamics in combination with two different force-length relations *F*_*ℓ*_(*ℓ*_CE_): either a parabola [7] or bell-shaped curves [11, 25]. For a given optimal CE length *ℓ*_CE,opt_ = 14.8 mm [26] representing a rat gastrocnemius muscle and three fixed exponent values *ν* = 2,3, 4 in Hatze's activation dynamics (all other parameters as given in Section 2), we thus determined Hatze's constant *ρ*_0_ and the width parameters of the two different force-length relations *F*_*ℓ*_(*ℓ*_CE_) (WIDTH in Kistemaker et al. [7] and van Soest and Bobbert [9] and Δ*W*_asc_ = Δ*W*_*des*⁡_ = Δ*W* in Mörl et al. [25], resp.) by an optimisation approach. The objective function to be minimised was the sum of squared differences between the Δ*ℓ*_CE,opt,submax_ values as predicted by the model and as derived from experiments (see Table  2 in Kistemaker et al. [7]) over five stimulation levels *σ* = 0.55,0.28,0.22,0.17,0.08. Note that *γ* = *σ* applies in the isometric situation (see (2) and compare (3)). Further note that experimental data for muscle contractions at very low stimulation levels are missing in the literature so far: the lowest analysed level available for Kistemaker et al. [7] was *σ* = 0.08, that is, comparable to the second rows from top in Figures 2 and 3.

The optimisation results are summarised in Table 2. The higher the *ν* value, the smaller the optimisation error. The predicted width values WIDTH or Δ*W*, respectively, decrease along with the error. We would yet tend to exclude the case *ν* = 4 because the predicted width values seem unrealistically low when compared to published values from other sources (e.g., WIDTH = 0.56 [9], Δ*W* = 0.35 [25]). Furthermore, *ρ*_0_ decreases with *ν* using the parabola model for *F*_*ℓ*_(*ℓ*_CE_) whereas it saturates between *ν* = 3 and *ν* = 4 for the bell-shaped model. The bell-shaped model shows the most realistic Δ*W* in the case *ν* = 3 (Δ*W* = 0.32). Fitting the same model to other contraction modes of the muscle [25], a value of Δ*W* = 0.32 had been found. In contrast, when using the parabola model, realistic WIDTH values between 0.5 and 0.6 are predicted by our optimisation for *ν* = 2.

When comparing the optimised parameter values across all start values of the *F*_*ℓ*_(*ℓ*_CE_) widths, across all *ν* values, and across both *F*_*ℓ*_(*ℓ*_CE_) model functions, we find that the resulting optimal parameter sets are more consistent for bell-shaped *F*_*ℓ*_(*ℓ*_CE_) than for the parabola function. The bell-shaped force-length relation gives generally a better fit. For each single *ν* value, the corresponding optimisation error is smaller when comparing realistic, published WIDTH and Δ*W* values that may correspond to each other (WIDTH = 0.56 [9] and Δ*W* = 0.35 [25]). Additionally, the error values from our optimisation are generally smaller than the corresponding value calculated from Table  2 in Kistemaker et al. [7] (0.23 mm).

In a nutshell, we would say that the most realistic model for the isometric force *F*_isom_ at submaximal activity levels is the combination of Hatze's approach for activation dynamics with *ν* = 3 and a bell-shaped curve for the force-length relation *F*_*ℓ*_(*ℓ*_CE_) with *ν*_asc_ = 3. As a side effect, we predict that the parameter value *ρ*_0_, being a weighting factor of the first addend in the compact formulation of Hatze's activation dynamics (5), should be reduced by about 40% (*ρ*_0_ = 3.25 · 10^4^ L/mol) as compared to the value originally published in Hatze [13] (*ρ*_0_ = 5.27 · 10^4^ L/mol).

### 7.4. A Generalised Method for Calculating Parameter Sensitivities

The findings in the last section were initiated by thoroughly comparing two different biomechanical models of muscular activation using a systematic sensitivity analysis as introduced in Dickinson and Gelinas [17] and Lehman and Stark [2], respectively. Starting with the latter formulation, Scovil and Ronsky [1] calculated specific parameter sensitivities for muscular contractions. They applied three variants of this method.

Method 1 applies to state variables that are explicitly known to the modeller as in, for example, an eye model [2], a musculoskeletal model for running that includes a Hill-type muscle model [1], or the activation models analysed in our study. Scovil and Ronsky [1] calculated the change in the value of a state variable averaged over time per a finite change in a parameter value, both normalised to each of their unperturbed values. They thus calculated just one (mean) sensitivity value for a finite time interval (e.g., a running cycle) rather than time-continuous sensitivity functions.

Method 2: whereas Dickinson and Gelinas [17] and Lehman and Stark [2] had introduced the full approach for calculating such sensitivity functions, Scovil and Ronsky [1] distorted this approach by suggesting that the partial derivative of the right hand side of an ODE, that is, of the* rate of change* of a state variable, with respect to a model parameter would be a “model sensitivity.” The distortion becomes explicitly obvious from our formulation: this partial derivative is just one of the two addends that contribute to the rate of change of the sensitivity function (10), rather than defining the sensitivity of the state variable itself (i.e., the solution of the ODE) with respect to a model parameter (8).

Method 3: Scovil and Ronsky [1] had also asked for calculating the influence of, for example, a parameter of the activation dynamics (like the time constant) on an arbitrary joint angle, that is, a variable that quantifies the overall output of a coupled dynamical system. Of course, the time constant does not explicitly appear in the mechanical differential equation for the acceleration of this very joint angle, which renders applicability of method 2 impossible. The conclusion in Scovil and Ronsky [1] was to apply method 1. Here, the potential of our formulation comes particularly to the fore. It enables calculating the time-continuous sensitivity of all components of the coupled solution, that is, any state variable *y*_*k*_(*t*). This is because all effects of a parameter change are in principle reflected within* any* single state variable, and the time evolution of a sensitivity according to (10) takes this into account.

In this paper, we have further worked out the sensitivity function approach by Lehman and Stark [2], presenting the differential equations for sensitivity functions in more detail to those modellers who want to apply the method. Furthermore, we enhanced the approach by Lehman and Stark [2] to also calculating the sensitivities of the state variables with respect to their initial conditions (17). This should be helpful not only in biomechanics but also, for example, in meteorology when predicting the behaviour of storms [28]. Since initial conditions are often just known approximately but start with the relative sensitivity values of 1, their influence should be traced to verify how their uncertainty propagates during a simulation. In the case of muscle activation dynamics, the sensitivities ${\tilde{S}}_{q_{Z,0}}$ and ${\tilde{S}}_{q_{H,0}}$, respectively, decreased rapidly to zero: initial activity has no effect on the solution very early before steady state is reached.

Furthermore, we included a second order sensitivity analysis which is not only helpful for an enhanced understanding of the parameter influence but also part of mathematical optimisation techniques [29]. The values of ${\tilde{R}}_{ijk}$ could be interpreted either as the relative sensitivity of the sensitivity ${\tilde{S}}_{ik}$ with respect to another parameter *λ*_*j*_ (and vice versa: ${\tilde{S}}_{jk}$ with respect to *λ*_*i*_) or as the curvature of the graph of the solution *y*_*k*_(*t*) in the *N* + *M*-dimensional solution-parameter space. The latter may help to connect the results to the field of mathematical optimisation in which the second derivative (Hessian) of a function is often included in objective functions to find optimal parameter sets.

### 7.5. Insights into Global Methods

Some additional conclusions can be drawn from global sensitivity analysis, in particular from comparing results in Section 6.3 to those based on local sensitivity analysis (Sections 6.1, 6.2, and 7.1).

For Zajac's activation dynamics, global analysis confirms local analysis in stating that there are no significant second or higher order sensitivities, with the slight exception of the phase of rapid change in activity after a step in stimulation. An experimenter who wants to measure the activation time constant *τ* can exclude influence from potentially slower deactivation processes (*β* < 1) by starting from high activity levels (Figure 2, bottom). It should yet be kept in mind that build-up of activity to the new level is not solely determined by *τ* but might be biased by other parameters than *τ* because TSI_*τ*_ peaks during the build-up phase (Figure 4, right).

In Hatze's activation dynamics, the higher order sensitivities play a clearly more significant role, even in the near-steady-state case (Figure 5: stronger deviation from 1 of both VBS and TSI). When arguing in terms of controllability of the models in Section 7.1, we speculated that Zajac's dynamics might be easier to control than Hatze's dynamics. Notwithstanding, Figure 5 shows that the stimulation is the most important control factor with even a higher importance than in Zajac's formulation.

At first sight unapparent, another result is the importance of *ρ*_*c*_. From a strictly local point of view we concluded that this parameter should have the same sensitivity as *σ* since they both are formally equivalent multipliers in Hatze's ODE (see relative sensitivities in Figure 3). However, the importance of *ρ*_*c*_ is significantly smaller than that of *σ*, in fact almost negligible. Their different global variabilities of values can give an explanation. The parameter *ρ*_*c*_ in the product *ρ*_*c*_ · *σ* ∈ [4; 11]×[0; 1] has a clearly lower relative variability than *σ*, measured in maximum percentage deviation from the respective mean value. The parameter *ρ*_*c*_ thus acts as an amplifier for *σ*. Similarly, the parameter *ν* has a relatively small variability throughout the literature. So, although its differential sensitivity is quite large, *ν* is found to have a low importance for the model output. For the latter fact there is yet another reason. In Section 6.2, we have emphasised that *ν* has a very changeful influence on solutions, depending on activity level. Additionally, its influence is highly dependent on other parameters like length *ℓ*_CE_ and *ρ*_*c*_ (see end of Section 7.1). Its strong influence in some situations and configurations is thus hidden by global averaging.

This demonstrates that the findings of global sensitivity analysis must be treated with caution because the whole dynamics of a system is condensed to a single average function per whole parameter range. Without local analyses of the solution space as exemplified in Sections 6.1 and 6.2 crucial features of its topology might be lost when solely relying on global analysis.

## Figures and Tables

**Table 1:** Lower and upper bounds for the parameter choices in both Zajac's and Hatze's models of activation dynamics.

Parameter	*β*	*ℓ*_CErel_	*ℓ*_*p*_	*m*	*ν*	*q*_0_	*q*_*Z*,0_, *q*_*H*,0_	*ρ*_*c*_	*σ*	*τ*
Lower bound	0.1	0.4	2.2	3	1.5	0.001	0.01	4	0	0.01
Upper bound	1	1.6	3.6	11	4	0.05	1	11	1	0.05

**Table 2:** Parameters minimising the sum over five submaximal stimulation levels *γ* = *σ* = 0.55,0.28,0.22,0.17,0.08 of squared differences between shifts in optimal CE length Δ*ℓ*_CE,opt,submax_(*γ*) (Δ*l*_MA,opt_ by Roszek et al. (1994) [27] in the third column of Table 2 in Kistemaker et al. [7]) at these levels predicted by the model with the isometric force *F*_isom_(*q*, *ℓ*_CE_) = *F*_max⁡_ · *q*(*γ* = *σ*, *ℓ*_CE_) · *F*_*ℓ*_(*ℓ*_CE_) and by experiments; simulated data represent a rat gastrocnemius muscle with an optimal CE length *ℓ*_CE,opt_ = 14.8 mm [26]; start value of *ρ*_0_ was 6.0 · 10^4^ L/mol; the exponents of the bell-shaped force-length relations *F*_*ℓ*_(*ℓ*_CE_) were fixed according to Mörl et al. [25] (*ν*_asc_ = 3, *ν*_*des*⁡_ = 1.5); the corresponding width values in the ascending and descending branch were assumed to be equal: Δ*W*_asc_ = Δ*W*_*des*⁡_ = Δ*W*; van Soest and Bobbert [9] and Kistemaker et al. [7] used a parabola for *F*_*ℓ*_(*ℓ*_CE_); for all other model parameters see Sections 7.3 and 2; optimisation was done by *f*minsearch (Nelder-Mead algorithm) in MATLAB with error tolerances of 10^−8^; *error* is the square root of the above-mentioned sum divided by five; corresponding error value given in Table 2 in Kistemaker et al. [7] was 0.23 mm.

*ν*	Bell-shaped [11, 25]			Parabola [7, 9]		
	Δ*W*_start_ = 0.25			WIDTH_start_ = 0.46		
	Δ*W* []	*ρ*_0_ [10^4^ L/mol]	*error* [mm]	WIDTH []	*ρ*_0_ [10^4^ L/mol]	*error* [mm]

2	0.46	3.80	0.08	0.63	8.78	0.10
3	0.32	3.25	0.05	0.41	5.45	0.07
4	0.26	3.20	0.02	0.34	4.60	0.05

	Δ*W*_start_ = 0.35			WIDTH_start_ = 0.56		
	Δ*W* []	*ρ*_0_ [10^4^ L/mol]	*error* [mm]	WIDTH []	*ρ*_0_ [10^4^ L/mol]	*error* [mm]

2	0.45	3.80	0.07	0.53	6.92	0.11
3	0.32	3.30	0.05	0.41	5.67	0.07
4	0.26	3.20	0.02	0.34	4.55	0.05

	Δ*W*_start_ = 0.45			WIDTH_start_ = 0.66		
	Δ*W* []	*ρ*_0_ [10^4^ L/mol]	*error* [mm]	WIDTH []	*ρ*_0_ [10^4^ L/mol]	*error* [mm]

2	0.45	3.78	0.07	0.55	7.35	0.11
3	0.32	3.25	0.05	0.41	5.35	0.07
4	0.26	3.20	0.02	0.34	4.56	0.05

## Symbols

*ℓ*_CE_: Contractile element (CE) length; value: time-depending
${\dot{\ell }}_{\text{CE}}$: Contraction velocity; value: first time derivative of *ℓ*_CE_
*ℓ*_CE,opt_: Optimal CE length; value: muscle-specific
*ℓ*_CErel_: Relative CE length; value: *ℓ*_CErel_ = *ℓ*_CE_/*ℓ*_CE,opt_ (dimensionless)
*F*_max⁡_: Maximum isometric force of the CE; value: muscle-specific
*σ*: Neural muscle stimulation; value: time-depending; here: a fixed parameter
*q*: Muscle activity (bound Ca^2+^-concentration); value: time-depending
*q*_0_: Basic activity according to Hatze [13]; value: 0.005
*q*_*H*_: Activity according to Hatze [6]; value: time-length-depending
*q*_*H*,0_: Initial condition for Hatze's activation ODE; value: mutable
*q*_*Z*_: Activity according to Zajac [5]; value: time-depending
*q*_*Z*,0_: Initial condition for Zajac's activation ODE; value: mutable
*τ*: Activation time constant in Zajac [5]; value: here: 1/40 s
*τ*_deact_: Deactivation time constant in Zajac [5]; value: here: 1/40 s or 3/40 s
*β*: Corresponding deactivation boost [5]; value: *β* = *τ*/*τ*_deact_
*ν*: Exponent in Hatze's formulation; value: 2 or 3
*m*: Activation frequency constant in Hatze [6]; value: range: 3.67,…, 11.25 (1/s); here: 10 (1/s)
*c*: Maximal Ca^2+^-concentration in Hatze [24]; value: 1.37 · 10^−4^ mol/L
*γ*: Representation of free Ca^2+^-concentration [6, 13]; value: time-depending
*ρ*: Length dependency of Hatze [24] activation dynamics; value: *ρ*(*ℓ*_CErel_) = *ρ*_*c*_ · ((*ℓ*_*ρ*_ − 1)/(*ℓ*_*ρ*_/*ℓ*_CErel_ − 1))
*ℓ*_*ρ*_: Pole in Hatze's length dependency function; value: 2.9
*ρ*_0_: Factor in van Soest [8], Hatze [6]; value: 6.62 · 10^4^ L/mol (*ν* = 2) or 5.27 · 10^4^ L/mol (*ν* = 3)
*ρ*_*c*_: Merging of *ρ*_0_ and *c*; value: *ρ*_*c*_ = *ρ*_0_ · *c*; here: 9.10 (*ν* = 2) or 7.24 (*ν* = 3)
Λ: Model parameter set; value: Λ = {*λ*_1_,…, *λ*_*n*_}.
