# Corrigendum to “Comparative Sensitivity Analysis of Muscle Activation Dynamics”

**DOI:** 10.1155/2017/6752731

**Published:** 2017-09-20

**Authors:** Robert Rockenfeller, Michael Günther, Syn Schmitt, Thomas Götz

**Affiliations:** ^1^Institut für Mathematik, Universität Koblenz, 56070 Koblenz, Germany; ^2^Institut für Sport- und Bewegungswissenschaft, Universität Stuttgart, Allmandring 28, 70569 Stuttgart, Germany; ^3^Institut für Sportwissenschaft, Lehrstuhl für Bewegungswissenschaft, Friedrich-Schiller-Universität, Seidelstraße 20, 07749 Jena, Germany; ^4^Stuttgart Research Centre for Simulation Technology, Pfaffenwaldring 7a, 70569 Stuttgart, Germany

We provide a comment to our paper “Comparative Sensitivity Analysis of Muscle Activation Dynamics,” Computational and Mathematical Methods in Medicine (2015), 16 pages, Article ID 585409, DOI 10.1155/2015/585409 [1], where we stated an erroneous form of Hatze's activation dynamics that is not applicable to non-steady-state muscle processes. However, as we only considered steady-state situations, all results and consequences still hold true. The authors would like to apologize for any inconvenience caused.

In his consecutive work [2–4], Hatze introduced the dynamics of changes in activity *q* (activation dynamics) for skeletal muscle fibers in response to neural stimulation *σ* as a multilevel process, with *γ* being the relative free calcium ion concentration and *ℓ*_CE_ the length of the contractile element (CE). In [4, Eqns. 3.27, 3.29, and 3.30], this process is summarized as follows:

(1) γ˙=m·σ−γ,γ0=γ0,ρlCE=ρc·lρ−1lρ·lCE,opt/lCE−1,qlCE,γ=q0+ρlCE·γν1+ρlCE·γν.

In our main article [1, Eqn. (5)], we had reformulated the above equation system (1) as

(2) q˙=ν·m1−q0·σ·ρlCE·1−q1+1/ν·q−q01−1/ν−1−q·q−q0,

in an effort to eliminate the state variable *γ* in favor of *q*. However, the specific formulation in (2) holds only true in the steady-state case ${\dot{\ell }}_{CE}=0$. This is because the transformation [5, Eqns. 3.21–3.24] was erroneously done by

(3) $$\begin{array}{c} \dot{q}=\frac{\partial q}{\partial \gamma }\dot{\gamma } \end{array}$$

rather than properly taking the total derivative

(4) $$\begin{array}{c} \dot{q}=\frac{\partial q}{\partial \gamma }\dot{\gamma }+\frac{\partial q}{\partial l_{CE}}{\dot{l}}_{CE} \end{array}$$

for the total time derivative of *q*.

In our framework only steady-state muscle conditions were investigated; that is, ${\dot{\ell }}_{CE}=0$, such that the second term of the right hand side in (4) vanishes. Hence, the situation from (2) holds throughout the article. In non-steady-state isometric contractions, this second term seems to be of reversed sign to the first, but with a considerably smaller absolute value; compare [6].
